# The Characteristics of Heterosexual STD Clinic Attendees Who Practice Oral Sex in Zhejiang Province, China

**DOI:** 10.1371/journal.pone.0067092

**Published:** 2013-06-25

**Authors:** Qiaoqin Ma, Xiaohong Pan, Gaofeng Cai, Jiezhe Yan, Yun Xu, Masako Ono-Kihara, Masahiro Kihara

**Affiliations:** 1 Department of HIV/STD control, Center for Disease Control and Prevention of Zhejiang Province, Hangzhou, PR China; 2 Department of Global Health and Socio-epidemiology, Kyoto University School of Public Health, Kyoto, Japan; Alberta Provincial Laboratory for Public Health/University of Alberta, Canada

## Abstract

**Background:**

The characteristics of heterosexual attendees who visit sexually transmitted disease (STD) clinics and practice oral sex have not been revealed in China. This information is important for the development of targeted STD prevention programmes for this population.

**Study Design:**

A self-administered questionnaire survey with a cross-sectional design was administered to consecutive attendees at four STD clinics in Zhejiang Province, China, between October and December in 2007. Demographic, psychosocial, and behavioural factors associated with oral sex over a lifetime were identified using univariate and multivariate analyses.

**Results:**

Of the 872 attendees, 6.9% engaged in oral sex over their lifetimes. Of the oral-sex group, 96.6% also engaged in vaginal sex. The correlates for oral sex over a lifetime as determined by the multivariate analysis were high income (odds ratio [OR] = 2.53, 95% confidence interval [CI] 1.39–4.59), high human immunodeficiency virus (HIV)-related knowledge (OR = 2.71, 95% CI 1.26–5.81), early sex initiation (OR = 2.42, 95% CI 1.37–4.27), multiple sexual partners (OR = 3.09, 95% CI 1.58–6.06), and sexually active in the previous 6 months (OR = 7.73, 95% CI 1.04–57.39).

**Conclusions:**

Though the prevalence of oral sex is low, the heterosexual STD clinic attendees practicing oral sex was found to have higher risks associated with STD/HIV transmission than those not. Behavioural and medical interventions conducted by clinicians in Chinese STD clinics should take into account the characteristics and related risks of those who practice oral sex.

## Introduction

Oral sex is more risky than people think. Oral sex can transmit sexually transmitted infections (STDs), including herpes simplex viruses, human papilloma viruses (HPV), syphilis, gonorrhoea, *Neisseria meningitidis, Chlamydia*, and chancroid [1]–[4]. STDs in the oral cavity can be asymptomatic or subclinical, and can be mistaken for ulcers or herpes, thus delaying curative treatment and allowing ongoing transmission. Oral STDs can also damage the mucosal surfaces of the oral cavity, and unprotected exposure, especially in the presence of lesions, can increase the risk of human immunodeficiency virus (HIV) transmission [1], [2], [4], [5]. Although the oral transmission of HIV is infrequent and substantially lower than through vaginal and anal intercourse, oral exposure to HIV was identified as an independent risk factor for acquiring HIV [6]–[8].

Data from the United States and Great Britain indicate that the percentage of men and women who engage in heterosexual oral sex has increased [9]–[13]. Over 80% of 15–44-year-olds in the United States reported ever having oral sex [11], while over 75% of 16–44-year-olds reported having oral sex in the past year in Great Britain [12], [13]. In Japan, the proportion of female STD clinic attendees practicing unprotected oral sex with regular and casual partners was 84% and 28%, respectively; the respective percentages were 55 and 2% for females in the general population [14]. A few studies have indicated that oral sex is associated with multiple sexual partnerships, having a non-monogamous sex partner [15], [16], much lower condom use when having oral sex compared to vaginal sex [15], [17], [18], an oral STD diagnosis [15], and an increased likelihood of contracting multiple STDs [16].

In China, few studies have examined the characteristics of individuals who practice oral sex and the risks of oral sex, even for high-risk populations. This is excluding reports on homosexual men, which suggest that the prevalence of oral sex is over 70% [19]–[21]. Given the possible increasing prevalence of oral sex among heterosexual partners in China due to rapid socioeconomic development, and associated negative health outcomes of oral sex, it is important to examine this behaviour in high-risk populations. Therefore, this study determined the prevalence and correlates of heterosexual oral sex in STD clinic attendees to understand the epidemiology and risks of this type of sexual behaviour.

## Methods

### Participants and Data Collection

In China, people who are concerned about current or possible STD problems will visit STD clinics for examination and treatment. The participants in this study were derived from a cross-sectional survey conducted at four STD clinics in Zhejiang Province, Eastern China, between October and December in 2007. Zhejiang Province is one of the most economically developed areas in China and is reported to have the highest incidence of syphilis and gonorrhoea in China [22], [23]. In 2007, Zhejiang Province established 12 HIV surveillance sentinels at STD clinics to collect basic information including HIV prevalence, demographic data (four questions), sexual behaviours in the last 3 months (three questions), drug use (one question), and blood plasma donation (one question) from April to June. Of these 12 STD clinics, 4 clinics located in the east, west, north, and middle area of the province agreed to participate in this study, the remaining eight declined participation due to a lack of interest in this research after review of the study protocol. Analysis was performed to compare the HIV prevalence and the attendees’ demographic characteristics in terms of gender, age, marital status, and residence among the four STD clinics and the remaining eight non-participating clinics. The analysis revealed that significant difference was not shown for HIV prevalence (0.40% versus 0.23%) and these demographic characteristics were generally evenly distributed between the two groups.

The research method has been introduced somewhere [24]. All sexually active attendees visiting these STD clinics for STD diagnosis and treatment who were >14 years of age were recruited to the study. Those attendees who were not sexually active, unwilling to participate in the study, had a language barrier, or who visited clinics for general skin diseases were excluded. During the study period, 1187 individuals (721 males, 466 females) visited the clinics for STD problems and 935 (601 males, 334 females) agreed to participate in the research. The response rate was 83.4% for males, 71.7% for females, and 78.8% overall. Of the 935 respondents, 908 responded validly. Of these, 16 of 586 males and 6 of 322 females had ever had sex with a same-sex partner. To avoid confounding heterosexual oral sex with same-sex oral sex, we excluded these 22 individuals from further analyses, resulting in a sample size of 886. Since the purpose of this study is to explore the characteristics of those attendees who practice oral sex, 14 attendees who did not answered the question regarding the types of sexual intercourse they performed over their lifetimes were not included in the analysis, therefore the final sample size is 872.

The questionnaire used in this study was developed after reviewing that used for HIV surveillance at the STD clinics at that time, the domestic and international literature, then modified after repeated discussion among the research team and doctors/nurses at the clinics. The final questionnaire had five sections consisting of 7, 10, 21, 8, 5 questions, respectively. The questionnaires were anonymous and self-administered by the STD clinic attendees. A nurse or doctor, who was trained by the research team prior to the study, explained the research and questionnaire to the participants. There was no incentive provided to the participants.

### Ethical Considerations

All attendees of the four clinics who met the recruitment criteria and adolescent attendees’ guardian were informed of the study purpose and method, and that participant privacy and confidentiality would be strictly protected, and whether participating in this study was at their discretion. They were also explained that filling in the questionnaire were regarded as that they understand and accept the survey. The above information was also printed at the beginning of the questionnaire. Since the research instrument was a questionnaire, those attendees including the guardian for adolescent attendees who gave verbal consent were given the survey after being documented in a register book.

Those responsible for institutional review at Zhejiang Province’s Center for Disease Control and Prevention and the four STD clinics approved the study protocol and consent procedure. The four clinics are situated in Haining city of Jiaxing prefecture, Jiangshan city of Quzhou prefecture, Deqing county of Huzhou prefecture, Yongkang city of Jinhua prefecture.

### Measures

Those attendees who engaged in any oral, anal, or vaginal sex were defined as sexually active. Oral sex was described as sexual activity involving contact between an attendee's mouth and his or her partner's genital. The participants were divided into two groups, i.e., those who did and did not practice oral sex. The oral-sex group included those who engaged only in oral sex, those who engaged in oral and vaginal sex, and those engaged in oral, vaginal, and anal sex; the no-oral-sex group included those who engaged only in vaginal sex and those who engaged in both vaginal and anal sex.

The HIV-related knowledge scale included four statements: 1) reported HIV cases had increased rapidly in recent years in Zhejiang, 2) HIV is spread from the high-risk population to the general population through sexual intercourse, 3) STDs makes a person more vulnerable to HIV, and 4) the correct use of condoms can reduce the transmission of HIV. There were three possible responses to the four statements: correct”, “incorrect”, and “unsure”. The scores for this scale ranged from 0–4, and participants were categorized into three groups based on the frequency distribution of this scale with 4 reflecting a high level of knowledge, 0–1 reflecting a low level of knowledge, and 2–3 an intermediate level of knowledge. Cronbach’s alpha for the internal consistency of this scale was 0.826.

### Statistical Analysis

The data were analysed using SPSS for Windows (ver. 17.0; SPSS, Chicago, IL). Initial analyses were conducted to describe the frequency and prevalence of various types of sex. The main study analyses compared participants who reported ever engaging in oral sex with participants who did not report oral sex activity over a lifetime in terms of demographic characteristics, history of sexual behaviour, reported STD history, and STD-related symptoms. We also compared the groups with respect to HIV-related knowledge, and STD and HIV risk perception.

The type of sexual intercourse over a lifetime was used as the dependent variable in the logistic regression analysis. Logistic regressions compared participants who did and did not engage in oral sex over a lifetime for different factors. Finally, variables that were significant in the univariate analyses, other than the number of sexual partners and condom use during the previous 6 months, were entered into a multivariate backward stepwise logistic regression model with a *P-*value >0.10 as the criterion for removing a variable, to examine independent factors associated with oral sex. All results were reported as odds ratios (OR) and 95% confidence intervals (95% CI), and were considered significant when *P*<0.05.

## Results

### Type of Sex Practiced by the Participants

Of the 872 attendees, 60 had engaged in oral sex at some time point (6.9% of total, 8.3% of males, and 4.2% of females), while 812 (93.1%) had not. Of the oral-sex group, 2(3.3%) performed only oral sex, 53(88.3%) engaged in both oral and vaginal sex, and 5(8.3%) engaged in oral, vaginal, and anal sex. Of the no-oral-sex group, 808(99.5%) engaged only in vaginal sex, while 4(0.5%) engaged in vaginal and anal sex.

### Demographic Factors Associated with Practicing Heterosexual Oral Sex

In the oral-sex group, 78.3% were male; the rate was 63.7% in the no-oral-sex group (Table 1). For those who performed oral sex and those who not, respectively, 51.7% versus 39.4% were les than 30 years older, 55.0% versus 69.5% were married, 80% versus 71.4% were local resident, 53.3% versus 66.0% didn’t get high school education, 36.7% versus 61.0% earned an income less than 2000 Yuan per month, 26.7% versus 54.6% were unemployed or a peasant.

**Table 1:** Demographic characteristics of STD clinics attendees who did and did not practice oral sex.

		Oral-sex group		No-oral-sex group			
Characteristics	Subgroups	n	(%^a^)	n	(%)	OR (95% CI)^b^	*P*-value
Gender	Female^c^	13	(21.7)	295	(36.3)	1	
	Male	47	(78.3)	517	(63.7)	2.06(1.10–3.88)	0.024
Age	<30^c^	31	(51.7)	320	(39.4)	1	
	30–39	17	(28.3)	258	(31.8)	0.68(0.37–1.26)	0.218
	≥40	12	(20.0)	234	(28.8)	0.53(0.27–1.06)	0.070
Marriage	Unmarried^c^	26	(43.3)	238	(29.3)	1	
	Married	33	(55.0)	564	(69.5)	0.54(0.31–0.92)	0.022
Residence	Locally^c^	48	(80.0)	580	(71.4)	1	
	Other area	12	(20.0)	200	(24.6)	0.73(0.38–1.39)	0.334
Education	Below high school^c^	32	(53.3)	536	(66.0)	1	
	High school and over	27	(45.0)	270	(33.3)	1.68(0.98–2.85)	0.058
Income	≤2000^c^	22	(36.7)	495	(61.0)	1	
	>2000	35	(58.3)	228	(28.1)	3.45(1.98–6.02)	0.000
Occupation	Unemployed/peasant^c^	16	(26.7)	443	(54.6)	1	
	Employed by government	17	(28.3)	132	(16.3)	3.57(1.75–7.25)	0.000
	Other	27	(45.0)	229	(28.2)	3.26(1.72–6.18)	0.000

a The percentage of attendees may not add up to 100% due to non-response for some items.

b OR: unadjusted odds ratio; CI: confidence interval.

c Reference category.

Table 1 also showed that practitioners of heterosexual oral sex were more likely to be male (OR = 2.06, 95% CI 1.10–3.88), less likely to be married (OR = 0.54, 95% CI 0.31–0.92), more likely to have earned an income >2000 RMB per month (OR = 3.45, 95% CI 1.98–6.02), more likely to have been employed by the government (OR = 3.57, 95% CI 1.75–7.25), and more likely to have worked in sectors other than being unemployed/a peasant or employed by the government (OR = 3.26, 95% CI 1.72–6.18) as compared with those who did not practice oral sex. The practitioners of oral sex did not differ significantly from non-practitioners in terms of age, residence, and education.

### Psychosocial and Behavioural Factors Associated with Engaging in Heterosexual Oral Sex

Table 2 indicates that the practitioners of heterosexual oral sex had significantly more knowledge about HIV (OR = 4.05, 95% CI 1.88–8.75 for intermediate score group, OR = 2.47, 95% CI 1.10–5.56 for high score group), and higher risk perception for HIV (OR = 2.54, 95% CI 1.02–6.31). In addition, they were more likely to have initiated sex before 20 years of age (OR = 2.86, 95% CI 1.68–4.86), experienced non-consensual sex (OR = 3.09, 95% CI 1.31–7.32), had a history of unwanted pregnancy (female or male’s female partner; OR = 1.84, 95% CI 1.08–3.15), reported multiple sexual partners (OR = 5.96, 95% CI 2.49–14.29 for >2 partners over lifetimes; OR = 2.21, 95% CI 1.08–4.53, OR = 10.56, 95% CI 5.26–21.18 for 2 and >2 partners, respectively, during the previous 6 months), reported sometimes/frequent condom use (OR = 2.52, 95% CI 1.46–4.35 for lifetime; OR = 2.10, 95% CI 1.20–3.67 for previous 6 months), and had been sexually active in the previous 6 months as compared with those who did not engage in oral sex.

**Table 2:** Psychosocial and sexual behaviours of STD clinics attendees who did and did not practice oral sex.

		Oral-sex group		No-oral-sex group			
Characteristics	Subgroups	n	(%^a^)	n	(%)	OR (95% CI)^b^	*P*-value
HIV knowledge scale	Low (0–1)^c^	9	(15.0)	302	(37.2)	1	
	Intermediate (2–3)	28	(46.7)	232	(28.6)	4.05(1.88–8.75)	0.000
	High (4)	19	(31.7)	258	(31.8)	2.47(1.10–5.56)	0.029
Oral sex transmits STDs	Unsure/no^c^	34	(56.7)	466	(57.4)	1	
	Possible	26	(43.3)	338	(41.6)	1.05(0.62–1.79)	0.845
STD risk awareness	Unsure/no^c^	31	(51.7)	526	(64.8)	1	
	Possible	25	(41.7)	276	(34.0)	1.54(0.89–2.66)	0.123
HIV risk awareness	Unsure/no^c^	54	(90.0)	777	(95.7)	1	
	Possible	6	(10.0)	34	(4.2)	2.54(1.02–6.31)	0.045
Age of first sex	≥20^c^	27	(45.0)	559	(68.8)	1	
	<20	33	(55.0)	239	(29.4)	2.86(1.68–4.86)	0.000
Length of sexual activity	1–6 years^c^	19	(1.7)	259	(31.9)	1	
	7–15 years	25	(41.7)	263	(32.4)	1.30(0.70–2.41)	0.413
	>15 years	16	(26.7)	276	(34.0)	0.79(0.40–1.57)	0.501
Experienced non-consensual	No^c^	53	(88.3)	772	(95.1)	1	
sex	Yes	7	(11.7)	33	(4.1)	3.09(1.31–7.32)	0.010
History of unwanted	No^c^	35	(58.3)	580	(71.4)	1	
pregnancy	Yes	25	(41.7)	225	(27.7)	1.84(1.08–3.15)	0.026
History of STDs^d^	No^c^	43	(71.7)	637	(78.4)	1	
	Yes	16	(26.7)	154	(19.0)	1.54(0.84–2.81)	0.159
Number of lifetime sex	1^c^	6	(10.0)	263	(32.5)	1	
partners	2	7	(11.7)	220	(27.2)	1.40(0.46–4.21)	0.555
	≥3	40	(66.7)	294	(36.4)	5.96(2.49–14.29)	0.000
Condom use lifetime	Never/rarely^c^	23	(38.3)	489	(60.2)	1	
	Sometimes/often	35	(58.3)	295	(36.3)	2.52(1.46–4.35)	0.001
	Always	2	(3.3)	23	(2.8)	1.85(0.41–8.32)	0.423
Sexually active in last 6	No^c^	1	(1.7)	125	(15.4)	1	
months	Yes	58	(96.7)	646	(79.6)	11.22(1.54–81.78)	0.017
STD-related symptoms^e^ in	No^c^	28	(46.7)	404	(49.8)	1	
last 6 months	Yes	32	(53.3)	392	(48.3)	1.18 (0.70–1.99)	0.542
Number of sex partners in	1^c^	17	(29.3)	416	(64.6)	1	
last 6 months	2	15	(25.9)	166	(25.9)	2.21(1.08–4.53)	0.030
	≥3	22	(37.9)	51	(7.9)	10.56(5.26–21.18)	0.000
Condom use in last 6	Never/rarely^c^	29	(50.0)	429	(66.4)	1	
months	Sometimes/often	26	(44.8)	183	(28.3)	2.10(1.20–3.67)	0.009
	Always	3	(5.2)	31	(4.8)	1.43(0.41–4.96)	0.572

a The percentage of attendees may not add up to 100% due to non-response for some items.

b OR: unadjusted odds ratio; CI: confidence interval.

c Reference category.

d STD mainly refers to gonorrhea, syphilis, chancroid, condyloma acuminatum, chlamydia, genital herpes, non-gonococcal urethritis, etc. in this research.

e STD-related symptoms refers to such symptoms as feeling pain or burning during one’s micturition, abnormal sexual organ secretion, anal ulcer or pain, skin damage or neoplasm on sexual organs, etc. in this research.

Performance of oral sex was not significantly associated with awareness that oral sex can transmit STDs, risk perception for contracting STDs, the length of sexual activity, reported history of a STD, or STD-related symptoms on the genitals in the previous 6 months as compared with participants who did not engage in this behaviour.

### Multivariate Model of Oral Sex

A multivariate logistic regression analysis showed that high monthly income (OR = 2.53, 95% CI 1.39–4.59), high HIV-related knowledge (OR = 2.71, 95% CI 1.26–5.81), early sex initiation (OR = 2.42, 95% CI 1.37–4.27), multiple sexual partners (OR = 3.09, 95% CI 1.58–6.06), and being sexually active in the previous 6 months (OR = 7.73, 95% CI 1.04–57.39) were significantly associated with oral sex (Table 3).

**Table 3:** Multivariate analysis predicting the practice of oral sex.

Variable	Subgroups	AOR (95% CI)^a^	*P*-value
Income	<2000^b^	1	
	≥2000	2.53(1.39–4.59)	0.002
Knowledge scale	Lower (0–1)^b^	1	
	Intermediate-high (2–4)	2.71(1.26–5.81)	0.011
Age of first sex	≥20^b^	1	
	<20	2.42(1.37–4.27)	0.002
Number of lifetime	<3^b^	1	
sex partners	≥3	3.09 (1.58–6.06)	0.001
Sexually active in last	No^b^	1	
6 months	Yes	7.73(1.04–57.39)	0.046

a AOR: adjusted odds ratio; CI: confidence interval.

b Reference category.

## Discussion

This study is among the first to examine the practices of oral sex among heterosexual STD clinic attendees in China. We found that among heterosexual participants, 8.3% of males and 4.2% of females practiced oral sex; this prevalence is consistent with a data from married women in urban area of one city, southern China [25]. The practice of oral sex is quite low among STD clinic attendees, a population at higher risk of STDs as compared with female STD clinic attendees in Japan [14] and the general populations in other developed countries [11]–[14]. Of those practicing oral sex, less than 4% practiced oral sex only, and the remaining practiced at least vaginal sex, indicating that most of these participants engaged in the 2 types of sex and were consequently at possibility of acquiring an STD by either vaginal intercourse or oral sex.

In this study, we define sexual activity as any oral, vaginal, or anal sex. Condom use was evaluated over a lifetime and during the previous 6 months. Unfortunately, we did not ask how often the subjects used condoms with each type of sexual behaviour. Consequently, we do not know how often oral sex was protected, or how the rate of protection differed from vaginal and anal sex. In our subjects, condom use was extremely low; 40∼50% of the participants in the oral-sex group never/rarely used condoms, while only 3% always used condoms throughout their lives and 5% always used condoms within the past 6 months. Other studies have shown that condom use is much lower during oral sex than during vaginal sex [15], [17], [18]. Therefore, it is reasonable to speculate that during oral sex, few of our participants were protected, leading to concern regarding the oral STD risk for this group.

Those who earn a high income are more likely to practice oral sex. Though data is not shown in the table, the further analysis revealed those who earned an average income >2000 Yuan per month, compared with those who earned ≤2000 Yuan, were more likely to be employed by the government (40.3% vs. 7.0%), less likely to be a peasant or unemployed (27.8% vs. 65.4%), more likely to have a high school or higher education (48.9% vs. 27.4%), more likely to have had sex with more than two partners (53.4% vs. 34.2%), more likely to report STD-related symptoms in the last half year (54.9% vs. 45.0%). However, there was no significant increase in those who always used condoms (3.8% vs. 2.5%). These data imply that our STD clinic attendees from a high social level are more sexually active and at higher risk for STD/HIV infection.

Not surprisingly, those practicing oral sex had more knowledge, given their higher socioeconomic status. Nevertheless, this knowledge did not translate into protective behaviour. Our findings indicate that those who practiced heterosexual oral sex were more likely to have sex with multiple sexual partners, be sexually active in the last 6 months? Multiple sexual partnerships and frequent sex are undoubtedly risky behaviours for STD/HIV infection if they are not effectively protected. Oral sex practitioners were also found to initiate sex at a younger age. The age at first sexual intercourse is a strong indicator of later adult sexual activity, and early sex initiation has been confirmed to be related to having more sexual partners, having sex more frequently [26]–[28], which is consistent with our findings, and being more likely to ever have been diagnosed with an STD. These findings suggest that those STD clinic attendees who practiced oral sex were more vulnerable to STD/HIV, the intervention program targeting them for STD/HIV prevention should centre on behaviour rather than knowledge, though we all understand that knowledge is important for a person to identify risky and protective behaviours, and the basis for behaviour change.

Although this study revealed an association between oral sex and risk factors for STD/HIV infection, there was no significant difference in the reported STD history over a lifetime or STD-related symptoms on the genitals in the last 6 months between the oral-sex and no-oral-sex groups. It appears that those STD clinic attendees who practise oral sex have no increased report of STD occurrence. Considering that there were no differences in condom use between the two groups in the multivariate analysis, and Chinese studies have revealed that those who practice oral sex are more likely to contract gonorrhoea, syphilis, condyloma acuminatum, etc [16], [29], further research therefore is needed to explore the difference of clinical and laboratory evidences between those who do and do not practice oral sex, and the causes behind them.

Previous research has shown that oral sex can transmit HIV [6]–[8] and specific STDs [1]–[4]. Only 43% of our subjects who engaged in oral sex believed that STDs could be transmitted through oral sex. Their risk perception for STDs and HIV was poor. More importantly, condom use during sex was extremely low. These data underscore the need to educate heterosexual STD clinic attendees practicing oral sex about their risk of orally transmitted STDs and HIV.

Our findings are limited by the cross-sectional design of the study, which does not permit us to ascertain cause-and-effect relationships. The participants may have differed from those who chose not to participate. The use of a consecutive sampling in 4 clinics over a limited period of time may result in selection bias, and limit the generalisability of our findings. Nevertheless, we believe that our findings have value as we compared the 908 participants in this study with 3072 participants from 12 HIV sentinels at STD clinics in 2007 for gender, age, marital status, and residence, and found that the distributions of them were all similar. Our findings may also be limited by the validity of the self-reported measures, as some participants may over-report socially desirable behaviours or under-report socially undesirable behaviours. Finally, those who practice insertive oral sex might be different from those who practice receptive oral sex to some extent. However, their exact difference is unknown in this study.

Our findings have important implications. First, although the reported prevalence of oral sex is low, and the risk of transmitting STDs via oral sex is lower than via vaginal intercourse, those heterosexual STD clinic attendees who practiced oral sex tended to have a profile placing them at higher risk for STD, including early sex initiation, multiple sexual partners, more frequent sex, and no increase of condom use even though they were more knowledged about HIV. Therefore, clinicians at STD clinics should note the characteristics and related risks of practitioners of oral sex. STD/HIV intervention targeting this population should focus more on sexual behaviour than knowledge. Second, the majority of our participants who practiced oral sex also performed vaginal intercourse and their rate of consistent condom use was extremely low. Therefore, this population is at a potential risk of contracting STDs or HIV through both vaginal and oral sex. STD clinics should highly recommend condom use during both vaginal and oral sex, in particular, emphasize that STD and HIV can be transmitted by oral sex as well. Third, heterosexual STD clinic attendees from a high socioeconomic level might be more sexually active and at greater risk. The characteristics of this group should be noted at STD clinics. In summary, oral sex practitioners among heterosexual STD clinic attendees are more sexually active and risky in comparison with that practising no-oral sex. STD clinic in China should take heed of the characteristics of this group, and implement appropriate behavioural and medical intervention for identifying and counselling them to reduce their sexual risks related to STD and HIV.
